# Microcellular Environmental Regulation of Silver Nanoparticles in Cancer Therapy: A Critical Review

**DOI:** 10.3390/cancers12030664

**Published:** 2020-03-12

**Authors:** Ganesan Raja, Yoon-Kwan Jang, Jung-Soo Suh, Heon-Su Kim, Sang Hyun Ahn, Tae-Jin Kim

**Affiliations:** 1 Department of Biological Sciences, Pusan National University, Pusan 46241, Korea; vraja.ganesan@pusan.ac.kr; 2 Integrated Biological Science, Pusan National University, Pusan 46241, Koreashahn970114@pusan.ac.kr (S.H.A.); 3 Institute of Systems Biology, Pusan National University, Pusan 46241, Korea

**Keywords:** silver nanoparticles, proteogenomics, metabolomics, genotoxicity, cancer therapy

## Abstract

Silver nanoparticles (AgNPs) play significant roles in various cancer cells such as functional heterogeneity, microenvironmental differences, and reversible changes in cell properties (e.g., chemotherapy). There is a lack of targets for processes involved in tumor cellular heterogeneity, such as metabolic clampdown, cytotoxicity, and genotoxicity, which hinders microenvironmental biology. Proteogenomics and chemical metabolomics are important tools that can be used to study proteins/genes and metabolites in cells, respectively. Chemical metabolomics have many advantages over genomics, transcriptomics, and proteomics in anticancer therapy. However, recent studies with AgNPs have revealed considerable genomic and proteomic changes, particularly in genes involved in tumor suppression, apoptosis, and oxidative stress. Metabolites interact biochemically with energy storage, neurotransmitters, and antioxidant defense systems. Mechanobiological studies of AgNPs in cancer metabolomics suggest that AgNPs may be promising tools that can be exploited to develop more robust and effective adaptive anticancer therapies. Herein, we present a proof-of-concept review for AgNPs-based proteogenomics and chemical metabolomics from various tumor cells with the help of several technologies, suggesting their promising use as drug carriers for cancer therapy.

## 1. Introduction

Engineered silver nanoparticles (AgNPs) have received a great deal of attention in mechanosensitive-based chemotherapies, immunotherapies, drug delivery mechanisms, and nanomedicine. Of late, due to the fact of their well-known bactericidal properties, AgNPs are used in marketed products as well as in a variety of medical applications [1,2,3,4,5]. Silver nanoparticles have been used to develop a bandage that can be used in a clinical department to reduce swelling, deliver medicine, and promote wound healing and environmental remediation [6]. Traditionally, AgNPs have been used in cosmetics, paints [7], household functions (e.g., air and water purification, food industry) [8,9], electronics, and textiles [10]. Silver nanoparticles and surface modified AgNPs have unique medicinal properties that can be exploited for use in bone cement [11] and catheters [12].

**Figure 1:**
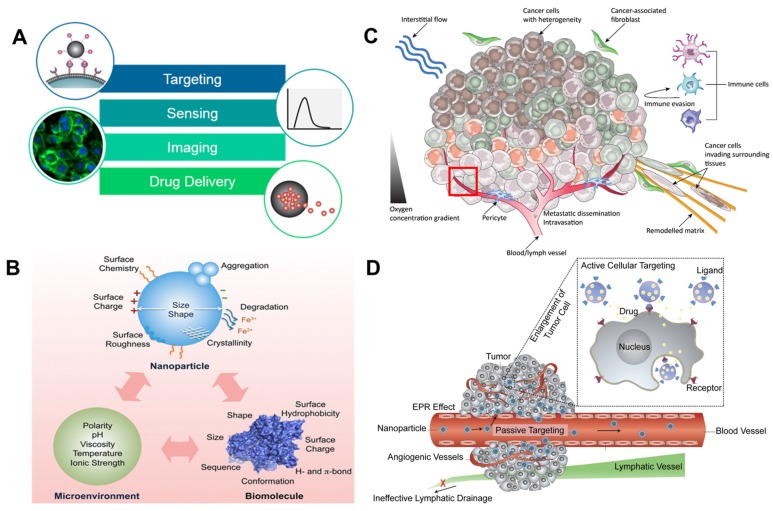
(**A**) Interface in cancer microenvironment: “magnified views” are schematic illustrations of main biomedical applications of NPs. Adapted from Reference [13], Copyright 2017 Wiley. (**B**) Biochemical and biophysical cues as well as important factors influencing NP–biomolecule interactions that regulate cell behavior. Reproduced with permission from Reference [16], Copyright 2014 American Chemical Society. (**C**) The plasticity and genetic heterogeneity of tumor cells cannot be analyzed using a solo method. The inner microenvironment synchronizes the movement, metastasis, and growth of tumor cells using spatiotemporally founded physiochemical and automatic signs. As an example, the interstitial flow triggers cell migration and tumor angio- and lymphangiogenesis. An extracellular matrix (ECM) interaction with cancer cell is the key technique of chemoresistance which is named cell-adhesion-mediated drug resistance (CAM-DR). In adjacent tissues, cancer-correlated fibroblast affects the renewal of the ECM, stroma, and collagen productions which directs the invasion of cancer cells into surrounding tissues. The pericyte acts as regulator of tumor angiogenesis which may be involved in metastasis. The red box below explains this in more detail. Adapted from Reference [32,33] Copyright 2019 Wiley. (**D**) The mechanisms of NPs can enter via active and passive cellular targeting and suppressing oncogenesis. The hypoxia-inducible factor (HIF)-facilitated chemoresistance and angiogenesis is established when a hypoxic environment is formed in the tumor area. When cell–cell communication occurs, cancer cells acquire their immune evasion qualities via various physiochemical kinetics. Active cellular targeting (box) is formed on the NPs’ surfaces with ligands that boost cell-specific detection and binding sites. Adapted with permission from Reference [14], Copyright 2013 American Chemical Society.

Nanoparticles (NPs) have extensive applications in medicine, because they can enter tumor cells, bind to the outer membrane of cells, act as extracellular sustained-release drug delivery, or be internalized into the tumor microenvironment. Nanoparticles have uses in biomedical applications; they can act as anticancer diagnostic agents by targeting, sensing, and imaging as well as drug delivery systems in conjunction with photodynamic therapy (PDT; Figure 1A) [13,14,15]. The intracellular microenvironment differs due to the various mechanical cues such as matrix stiffness, topography, shear stress, and mechanical stretching. Mechanical cues are regulated according to NPs’ size, aggregation, softness/stiffness, stability, and surface chemistry. These factors are important players in dynamic tumor microenvironments and cancer metastasis controls. Furthermore, it is important to identify specific microenvironmental responses that depend on the surface hydrophobicity, hydrogen bonds, π bonds, surface coating, stability, and treatment concentrations of AgNPs. The surface chemistry, polarity, degradation, and crystallinity of AgNPs alter the pristine materials shape, size, sequences, agglomeration property, and dissolving ratio in cells. The surface chemistry can precisely control and tailor the influences Ag^+^ ion releases, pH, ionic strength, temperature, and stability. It can be stored at 5 °C in dark for long-term stability. AgNPs in phosphate buffered saline (PBS) or colloidal solution has a huge impact on biological systems interactions which has been considerable interest. The surfactant pressure-area (π-A) of AgNPs is determined with the ζ-potential analysis (Figure 1B) [16,17,18]. This kind of heterogenous stimulation of tumor cells leads to DNA degradation which may induce toxicity. There are two possible molecular mechanisms through which AgNPs provide programmed cellular suicide or survival in defected situations. The first involves the dispersion and following delivery of silver ions (Ag^+^) [17]. The second involves the generation of reactive oxygen species (ROS) either by Ag^+^ or AgNPs. Ag^+^ ions possess a greater potential for toxicity than elemental Ag and AgNPs. However, reports of prior investigations that Ag^+^- and AgNPs-mediated oxidative stress are conflicting [3,17,19].

Moreover, with the help of various redox mechanisms, AgNPs have been shown to induce cell survival, anti-proliferation, cytotoxicity, and genotoxicity. According to redox metabolisms, proteogenomic changes in tumor microenvironments have revealed the metabolic conditions under which harmful effects and apoptosis can be identified [20,21]. Studies have shown that, upon exposure, AgNPs bioaccumulate in specific organs such as human liver cells. This has also been observed in lung cells via inhalation exposure [22,23]. Other studies have assessed the mechanobiological influence of AgNPs in fibroblasts and macrophages considered to further develop their potential applications in wound dressings, such as anti-infectives, and in disease management [24,25,26].

## 2. Biodistribution of AgNPs and Mechanical Cues Regulating Tumor Growth

In cellular microenvironments, AgNPs are biodistributed to the major sites of ROS production in the cell [27,28]. ROS are generated in various tumor cells due to the changes in pH which are linked to cellular stiffness. This stiffness determines cell differentiation that can induce cells to differentiate into human skin cells following AgNP exposure [29]. A possible transcription profiling in AgNPs- and Ag+-associated toxicity has been underlined in in vivo conditions such as zebrafish embryos. Hydrogen ions (H^+^) ions are abundant within mitochondria, where H^+^ efflux is the main event (i.e., proton motive force) in ATP synthesis (i.e., energy production) [30,31].

### 2.1. Heterogeneity of Cancer Cells

The genetic heterogeneity and plasticity of cancer cells cannot be studied using only a single-omic technique. We know that the inner microenvironment synchronizes the migration, metastasis, and growth of cancer cells using spatiotemporally formed biophysical, biochemical, and mechanical cues (Figure 1C). For example, the interstitial flow triggers cell migration and tumor angio- and lymphangiogenesis. The interaction of the extracellular matrix (ECM) with cancer cells is a major mechanism of chemoresistance. This is referred to as cell-adhesion-mediated drug resistance. In surrounding tissues, cancer-correlated fibroblasts affect the renewal of the ECM, which directs the invasion of cancer cells into surrounding tissues. The pericyte acts as a regulator of tumor angiogenesis which may be involved in metastasis (please refer to the detailed explanation in the red box below [32,33]. The molecular mechanisms of NPs allow them to enter cells via active and passive cellular targeting, leading to a suppression of oncogenesis. Low metabolism, hypoxia-inducible factor (HIF)-mediated chemoresistance, and HIF-mediated tumor angiogenesis occur when a hypoxic environment develops in the tumor mass. When cell–cell interactions occur, cancer cells acquire their immune evasion characteristics via various physiochemical kinetics. Active cellular targeting (box) is achieved when NP surfaces and ligands promote cell-specific recognition and binding (Figure 1D) [14].

### 2.2. Phagocytosis and ROS Generation

**Figure 2:**
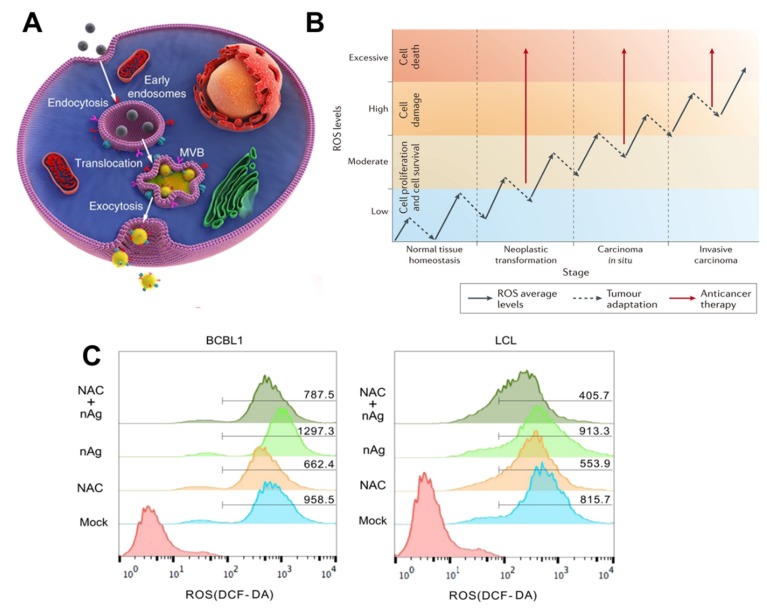
(**A**) Schematic illustration of Doxorubicin (DOX)-coated NPs endocytosed into tumor cells following incubation, then found in multivesicular bodies (MVBs) and autophagosomes. After MVBs or amphisomes fused with cellular membrane, DOX-coated NPs are exocytosed into extracellular space. Adapted from Reference [40], Copyright 2019 Nature. (**B**) A comparative analysis of ROS levels and oncogenesis at various periods. The cells undergo a radical growth of ROS volumes during the conversion from regular position to aggressive carcinomatous form (solid arrows). Still, those cancer cells can escape oxidative stress via boosting its innate antioxidant defense systems that will reduce ROS levels (dashed arrows). Subsequently, it here is recommended that we could force the accretion of too much ROS in tumor microenvironment that overpowers the antioxidant systems to induce apoptosis for chemotherapy medicines (solid red arrows). Reprinted with permission from Reference [36], Copyright 2019 American Chemical Society. (**C**) Cells individually treated with NAC, nAg (5 μg/mL), and nAg+NAC for 1 h were investigated via flow cytometric analysis. AgNP treatments significantly increased ROS levels. Adapted with permission from Reference [65], Copyright 2019 Springer Nature.

AgNPs enter cells via diffusion, phagocytosis, or endocytosis, and they can move towards the mitochondria, cytoplasm, nucleus, vesicles, etc. (Figure 2A). The mechanistic ROS act as the hallmark of cancer which could be associated with anti-proliferation metabolism. Translocation of AgNPs into the mitochondria, nucleus, and redox active organelles in the microenvironment is considered to result in the formation of ROS. In many tumor and non-tumor cell lines, ROS affect membrane disruption, mitochondrial damage, oxidative stress-related mRNA and DNA damage, and eventual cell death by apoptosis [20,21,22,23]. Many studies have shown the chemical redox activity of the tumor microenvironment in association with intrinsic oxidative damage-dependent pathways. Low concentrations of AgNPs trigger increases in antioxidants such as glutathione (GSH), GSH peroxidases (GPXs), and super oxidase dismutase (SOD). Moreover, decreases in lipid peroxidation have been shown by some researchers, whereas others have noted decreases in antioxidant levels in the presence of AgNPs [20].

Inside the cell, the AgNPs themselves or ionized Ag+ generate ROS, resulting in oxidative stress. In cellular microenvironments, radicals (i.e., superoxide, O_2_^-^; hydroxyl radical, OH; nitric oxide, NHO; organic radical, R^.^; peroxyl radical, ROO; alkoxy radical, RO; and nitrogen dioxide, NO_2_) and non-radicals (i.e., hydrogen peroxide, H_2_O_2_; singlet oxide, O_2_; ozone, O_3_; and peroxynitrile, ONOH^-^) may randomly increase oxidative stress/damage and biological disturbances [34]. This biochemical phenomenon leads to mechanobiological stress between the tumor and non-tumor cells (Figure 2B). AgNP-induced mechanistic oxidative stress is a possible mechanism responsible for controlling cellular processes in cancer [35,36].

Recent studies have shown that the expression of GSH and subunits of two GSH-synthesizing enzymes along with the associated mRNA expression are downregulated in human liver cells. These responses were accompanied by mitochondrial membrane disruption through the downregulation of Bcl-2 protein expression and concomitant upregulation of Bax protein expression [21,37]. The opening of the mitochondrial membrane is inhibited and stimulated by Bcl-2 and Bax, respectively. Their combined effects have been exploited for the delivery of cytochrome C into the cytosol which can initiate the activation of caspase 3 and caspase 9, leading to apoptosis [21,38]. Herein, we have detailed the redox activity of AgNPs as well as their role in cytotoxicity/genotoxicity-induced programed cell death and, subsequently, their utilization as a targeted therapy against tumor proliferation [39,40,41,42]. The summary of molecular effects, risk classification, and physiochemical properties of AgNPs are summarized in Table 1.

### 2.3. Biodistribution of AgNPs and Tumor Targeting

Many in vitro studies have been performed to investigate the microenvironmental mechanisms through which AgNPs induce genotoxicity or cytotoxicity [58,59,60]. In vivo studies have also been carried out to substantiate in vitro studies. The data from these studies have shown that ROS-dependent pathways play a significant role in AgNP cytotoxicity. In one study, the effects of different concentrations of AgNPs (26 mg kg^−1^, 52 mg kg^−1^, and 78 mg kg^−1^) were analyzed in Swiss albino mice at 24 h and 72 h [31]. Time- and dose-dependent DNA damage was detected in the liver cells and lymphocytes of the mice in the aforementioned study. Furthermore, a liver tissue sample was identified dose- and time-dependent apoptosis of the liver cells and necrosis in other regions [30,43,61].

In vitro, NPs have been shown to access the direct fraction of SKOV-3 tumor blood vessels in a time-dependent manner. The delivery efficiency and distribution of nanoparticles in whole solid tumors have also been reported. Similarly, another study confirmed the increased biomarkers of oxidative stress after treating Swiss albino mice with different doses of AgNPs over a 14-day period [43,59,62]. Subsequently, GSH levels in the blood of the treated mice decreased, indicating an increase in ROS in the blood. Conversely, the effect of AgNPs on tissue ROS levels varied depending on the environment. The DNA damage was significantly increased in the urine [62]. The production of ROS was used as a measure of cytotoxicity/genotoxicity, because it leads to changes in the expression of matrix metalloproteinases (MMPs), DNA damage, and cell death via apoptosis. The production of ROS can be confirmed using a fluorescence-based assay and through the independent introduction of two antioxidants, N-acetyl cysteine (NAC) and vitamin C. Interestingly, both NAC and vitamin C attenuate AgNP-induced ROS production, but only NAC prevents loss of MMPs, initiation of DNA damage, and apoptosis [63,64]. It has been proposed that NAC may act as an Ag+ scavenger which would suggest that the loss of MMPs, DNA damage, and apoptosis are, at least in part, due to the presence of ionic silver (Figure 2C) [25,65].

For medicinal purposes, these in vivo studies have significant implications for the application of AgNPs, and, as most of the deleterious effects of AgNPs were found at the highest concentrations, these studies have highlighted the importance of using appropriate AgNP doses to mitigate adverse toxicological effects [66,67,68]. To develop therapeutic drugs, the molecular mechanisms by which AgNPs may induce harmful effects must be fully understood. Polyvinyl pyrrolidone (PVP)-coated AgNPs have displayed amplified cytotoxic effects against six different cell lines from patients with acute myeloid leukemia (AML) compared to cells from healthy patients [25]. Accordingly, concentration-based cell viability was examined with three different sizes of AgNPs with no significant changes in the IC_50_ (IC_50_, ~4 μg/mL) for AML and healthy cells. At low AgNPs doses (~1 to 2 μg/mL), cell viability was actively reduced, more significantly in AML cells than in healthy cells which may be representative of the increased cytotoxicity and genotoxicity of AgNPs in AML cells [25]. Collectively, this is comparatively significant with other higher concentrations delivering AgNPs to cancer cells with high efficiency and efficacy [26].

### 2.4. Relative Tumor Thermal Therapies

**Figure 3:**
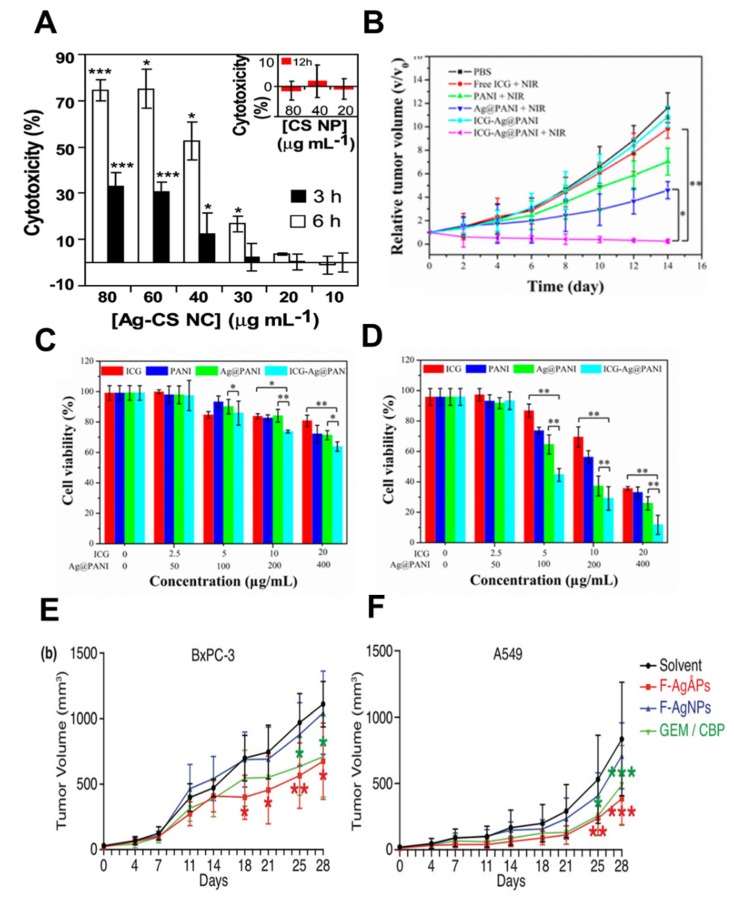
(**A**) The cytotoxicity comparison of AgNPs-chitosan nanocarrier (Ag-CS NCs) on HT29 cells, as calculated from the LDH assay. The cytotoxicity of CS NPs treatment is shown in the inset. Reprinted with permission from Reference [26], Copyright 2011 American Chemical Society. (**B**) The comparison of ani-tumor effects from mice carrying tumor-infused HeLa cells with different concentrations, monitored with and without NIR laser irradiation (808 nm, 1.0 W cm^2^). Cell viability in HeLa cells was calculated as a percentage with a variety of drugs such as indocyanine (ICG), polyaniline (PANI), Ag@PANI, and ICG-loaded PEgylation AgNPs core PANI nanocomposites (ICG-Ag@PANI) without (**C**) or with (**D**) NIR laser irradiation (808 nm, 1.0 W cm^−2^, 5 min). Reprinted from Reference [71], Copyright 2016 American Chemical Society. (**E**,**F**) The first-line chemotherapeutic drugs of fructose-Angstrom (Ang)-silver particles (F-AgAPs), F-AgNPs were intravenously treated on BxPC-3 pancreatic cancer and A549 lung cancer xenografts in nude mice. The tumor volume in mice was quantitatively decreased according to time scales. GEM: gemcitabine, CBP: carboplatin. Reprinted with permission from Reference [72], Copyright 2019 Wiley.

AgNP toxicity (cytotoxicity and genotoxicity) has been well studied. Dose-based AgNPs can act as biocompatible nanocarriers (NCs). More deeply, functionally modified AgNPs with chitosan NCs (Ag-CS NC) have been shown to exhibit increased genotoxicity, and cytotoxicity in human colon cancer cells with an IC_50_ of 0.33 μg/mL (Figure 3A). This cellular toxicity proceeds through an apoptotic pathway triggered by ROS production and mitochondrial dysfunction. In addition, the cytotoxic and genotoxic nature of AgNPs towards cancer cells combined into NCs, coupled with biocompatibility and targeted delivery potentiates the use of AgNPs as anti-tumor therapies [26]. AgNPs applied cell viability in different cancer and non-cancer cells are promising platforms to find the proliferation rate. The cell viability of AgNPs has been tested in normal and hypoxia environments [69,70].

Anti-tumor proliferation was assessed in HeLa tumor-bearing mice that were injected with various formulations of AgNPs as indicated, with or without subsequent NIR laser irradiation (808 nm, 1.0 W cm^–2^). The viability of HeLa cells incubated with various doses of free indocyanine (ICG), (polyaniline) PANI, Ag@PANI, and ICG-loaded PEgylation AgNPs core PANI nanocomposites (ICG-Ag@PANI), without (C) or with (D) subsequent NIR laser irradiation (808 nm, 1.0 W cm^–2^, 5 min) (Figure 3B–D) [71].

AgNPs are useful for the development of novel nanodrugs; they use a mechanobiological microenvironmental mechanism to cause apoptotic cell death via ROS-mediated pathways. The general understanding of the mechanosensitivity of AgNPs to biochemical pathways and small molecules has been demonstrated in well-controlled studies. Finally, to utilize AgNPs as direct therapeutic agents or as NCs, their properties must be carefully tuned [47]. A first-line chemotherapeutic drug, fructose-Angstrom (Ang)-silver particles (F-AgNPs) was intravenously administered in BxPC-3 pancreatic cancer and A549 lung cancer xenografts in nude mice (Figure 3E,F). As per this study, tumor growth was significantly suppressed in mice [72].

Due to the ROS overproduction, the antioxidant defense system along with associated lipids and proteins can collapse [25]. Mitochondria mostly release ROS following AgNPs exposure which can lead to oxidative stress and decreased expression of Bcl-2 which is also responsible for ROS generation, mechanisms of cytotoxicity, and, finally, apoptosis [43]. AgNPs (0 to 50 µg/mL)-induced redox-mediated cell viability experiments have shown that higher concentrations of AgNPs result in increased toxicity. Accordingly, it was concluded that delivery of a high dose of nanomaterials results in increased apoptosis compared to lower doses. Moreover, in the cellular microenvironment, AgNPs usually produce ROS after endocytosis into the cytoplasm [39]. When ROS quantitively increases, the gene ontology, protein modification, DNA repair, DNA replication, cell death, M phase, and RNA biosynthetic process changes. ROS can affect growth factor receptors (activator protein-1, AP-1; NF-kB) and induce oxidative DNA damage [44]. Specifically, the redox status of three genes (i.e., glutathione synthetase (Gss), glutathione peroxidase (Gpx), and thioredoxin reductase 1 (Txnrd)) in the cellular microenvironment were analyzed [45]. Additionally, the selective disruption of the mitochondrial respiratory chain by AgNPs was noted in fibroblast cells which may cause neurodegeneration [46].

The mechanobiology of oxidative stress-related genes (i.e., catalase; superoxide dismutase 1, SOD1; and glutathione peroxidase 1, GPx-1) upon exposure to pure AgNPs in human hepatoma cells has been studied; these genes have been found to be implicated in maintaining antioxidant defense capacity [39]. The mRNA levels of GPx1 and catalase were amplified. AgNPs and Ag+ ions were found to exhibit cytotoxicity and genotoxicity with a potential oxidative stress-related mRNA gene. Furthermore, the molecular activity of AgNPs may be different from that of Ag+ ions. Moreover, DNA breakage and their chemical compositions may lead to apoptosis [44,73,74,75].

### 2.5. Cell cycles and Cell Death

**Figure 4:**
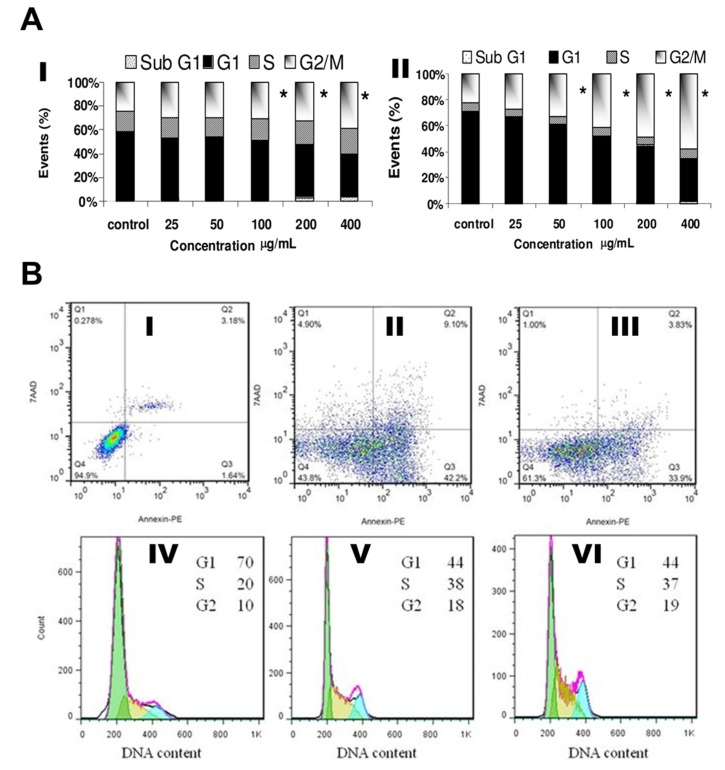
The condition of AgNP-treated cell cycles in U251 cells: (**A-I**) the gradual increase of the S/G2 stage. (**A-II**) the G2/M arrest in IMR-90 cells which may happens in a concentration (µg/mL)-dependent manner. All panels reprinted with permission from Reference [74], Copyright 2008 American Chemical Society. 2D density plots of panels (**B-I**, **II** and **III**) provide a fraction of early apoptotic, late apoptotic, and necrotic cell murder of MDA-MB-231 cells, respectively. Panel **(B-I)** exposures of the natural cells as controls, while panels (**B-II** and **B-III**) exhibit the cells with an LD_50_ of AgNPs and albumin-coated-AgNPs exposures, respectively. Necrosis and apoptosis were assessed with the use of 7-AAD and Annexin-V dyes in flow cytometry methods. Panels (**B-IV**, **V**, and **VI**) demonstrate the impact of albumin-coated-AgNPs exposures on the cell cycles. (**B-IV**) Untreated cells and (**B-V** and **B-VI**) tumor cells with AgNPs and albumin-coated-AgNPs exposures, respectively. The cells’ DNA content was plotted with help of flow cytometry. Adapted from Reference [79], Copyright 2017 Nature.

AgNPs have been shown to arrest cell cycle transitions such as gap1 (G1/G0), DNA synthesis (S), and gap2/mitosis (G2/M) in human glioblastoma cells (U251; Figure 4A-I) and in IMR-90 cells (Figure 4A-II) which cause severe G2/M arrest with repaired DNA damage. They were evaluated the G2/M cycle blocking with various concentration-dependent manner [76]. Similarly, density plots were constructed based on early apoptosis, late apoptosis, and necrotic cell death progression in MDA-MB 231 cells treated with AgNPs for 24 h. Increased necrosis and late apoptosis occurred upon exposure to uncoated AgNPs, compared to albumin-coated AgNPs (Figure 4BI–III). Therefore, albumin-coated AgNPs show promise as chemotherapeutic agents in the future [77,78,79].

Purine and pyrimidine bases are linked to several pathways that are damaged by AgNPs. These pathways may trigger metabolic transduction leading to DNA base pair impairments. It acts as a DNA backbone [47,48]. Moreover, mitochondrial activity is significantly affected and, as a result, the top 10 most down- and upregulated proteins are identified by 20 nm and 100 nm. Gene ontology has revealed the proteins involved in cell death, cell growth, antioxidant activity, and mitochondrial activity in LoVo cells [49]. NanoAg-induced oxidative-stress responses gene (i.e., mt-2A, HO-1, and hsp70) and protein homeostasis was estimated and quantified [80]. These genes were upregulated, which indicated apoptosis induction by ROS. Moreover, these genes are associated with the potential genotoxicity and cytotoxicity of AgNPs, leading to apoptosis-mediated cell death [50]. To maintain the total ROS under the toxicity threshold, the ROS generation and eradication ratio is important to maintain the equilibrium state of redox dynamics from the normal redox homeostasis to a new steady state.

The redox-sensitive transcriptional nuclear factors (e.g., nuclear factor kappa-light chain-enhancer, NF-κB; AP-1; nuclear factor erythroid-derived 2-like 2, Nrf2), expression of ROS-scavenging molecules (e.g., SOD and GSH), or changes in gene expression via complex metabolic metabolisms (e.g., genetic or epigenetic pathways) have been significantly reduced [39,80]. Additionally, catalase and SOD act as intercellular defensive enzymes. Glutathione acts primarily as an antioxidant scavenger that can bind and block the generation of ROS. Regarding non-cancer cell survival, the GSH-based antioxidant/ROS scavenger system is significant and acts as a dangerous defense system. Cancer cells are mostly allowed to escape oxidative stress and survive under high redox conditions [81].

## 3. AgNPs Currently Applied Proteogenomic Imaging and Drug Delivery

### 3.1. Drug Delivery and Theragnostics

**Figure 5:**
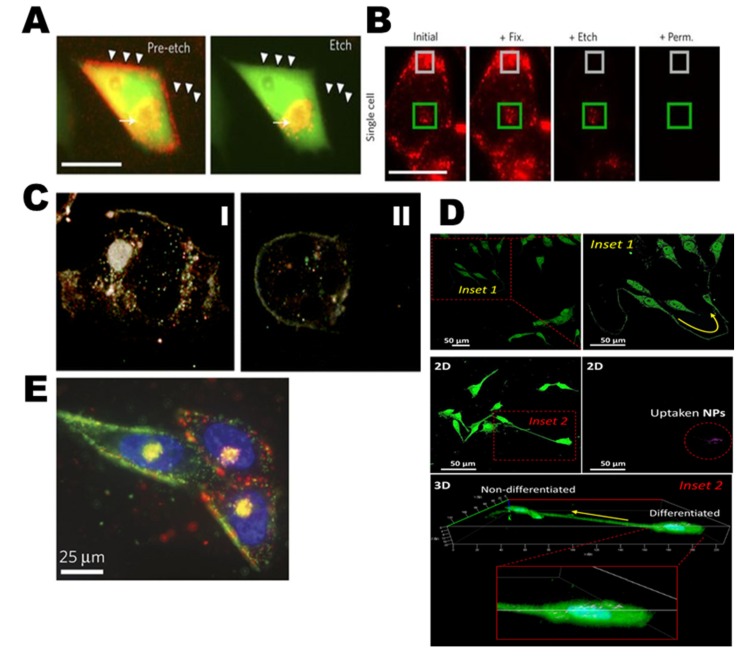
A schematic representation of the intracellular tracking, bioimaging, and drug delivery of AgNPs in the cancer microenvironment. (**A**) Bioimages of GFP-expressing PC-3 cells (green) when attaching and endocytosis of R-Ag-NA555 (red) has shown. The cell expresses the NRP-1 receptor. R-Ag-NA555 seems red when not correlated with the GFP in cells, and these were eliminated via etching (arrowheads). Cellular nuclei have been stained with a fluorescent dye (Hoescht blue). Yellow color signifies cellular correlation with R-Ag-NA555 due to the spatial overlap with the cellular environments, shown by the full arrows. (**B**) At the single-cell level, sequential bioimaging of fixing (+ Fix), etching (+ Etch), and permeabilization (+ Perm) in PC-3-GFP cells with exposure of R-Ag-NA555 have been analyzed. Drug delivery of internalized R-Ag-NA555 that were in cellular microenvironments (green boxes) and areas of attached but external particles (grey boxes) are exhibited. Reprinted with permission from Reference [82], Copyright 2014 Nature. (**C**) Optical bioimages of single cells show AgNPs accumulated in both the cytoplasm (**C-I**) and nuclei (**C-II**) of the cell. Adapted from Reference [83], Copyright 2010 Royal Society of Chemistry. (**D**) The two ways of cell-to-cell distinction after Ag@Bi2Se3/PVP-3WJ-RA/R transfection. The yellow arrows show the differentiation tracks; Inset 2 shows the 3D image of the cell-to-cell interaction along with a high magnitude image to display the NPs’ locations inside the differentiated cells. Panel reprinted with permission from Reference [100], Copyright 2019 American Chemical Society. (**E**) PPC-1 cells have been incubated with the mixture of green and red Ag-NA (AgNPs coated NeutrAvidin-PEG-thiol), holding Lyp-1 and RPARPAR peptides, respectively. The variation of attachment among the cells may expose receptor-specific binding with colocalization in endosomes. This may occur very near to the nucleus (Hoescht, blue). Reprinted with permission from Reference [82], Copyright 2014 Nature.

Tracking AgNPs within live PC-3 cells (green) are displayed and endocytosed in the materials that appear red on cellular membranes (Figure 5A,B). Nuclei are shown with R-Ag-NA555 (red) when not bounded with the GFP in cells. R-Ag-NA555 particles can incubate with single cell of PC-3-GFP and fixing (+Fix), etching (+Etch), and permeabilities (+Perm). The internalized and externalized particles are showed as green and gray box respectively [82]. The cytotoxic and genotoxic activity of AgNPs as well as their use as targeted biomarkers in different cancer cells lines are detailed in Table 2. Many studies have recently shown that different sizes of AgNPs induce toxicity by triggering the production of ROS. An in vitro study [83] has also shown that the accumulation of AgNPs into the nucleus (Figure 5C-I) and cytoplasm (Figure 5C-II) may promote ROS production that could stimulate cell death-regulating central mechanisms, such as p53, caspase-3, AKT signaling, inflammation (i.e., TNF-α, IL-1β, IL-6, IL-8. etc.) and p38 MAPK signaling apoptotic pathways [22,38,84]. Caspase-3 is responsible for cleaving other caspases. The cleavage of downstream effectors of caspase proteins has revealed that AgNPs significantly inhibit cancer cell proliferation by apoptosis [85].

Cell-to-cell differentiation with fabricated nano-biohybrid materials (bismuth selenide nanoparticles, Bi_2_Se_3_ NPs) is core-shelled with silver (Ag@Bi2Se3)) with transfection and is shown in Figure 5D including two-dimensional (2D) images of cell–cell interaction with 36 h Ag@Bi_2_Se_3_/PVP-3WJ-RA/R transfection (Inset 2). Moreover, with the help of 3D imaging techniques, AgNPs locations inside the differentiated cells and non-differentiated cells have been used to develop drug release and cell fate [100]. In Figure 5E, near the nucleus, the heterogenic binding pattern of green and red fluorescent Ag-NA are differentially targeted and multiplexed in PPC-1 cells with Ag-NA. Two different peptides have been applied which bind to different receptors in PPC-1 cells [82].

The signaling pathways of p53, Akt, and MAPK by AgNPs have also been investigated. Total phosphorylated Akt was found to be elevated after treatment with AgNPs which is a downstream target of P13K, and phosphorylated p38 MAPK protein expression was noticeably upregulated [38,85]. Additionally, HepG2 cells treated with AgNPs showed actively increased expression of the proapoptotic kinase, p38. Accordingly, inhibition of PI3K or p38 MAPK signaling enhanced nano-Ag-induced cell death. Therefore, these data support the ROS-based anticancer activity of nano-Ag particles [38]. The mitochondrion plays a central role in apoptosis signal developments. The mechanical effects of AgNPs may affect the expression of genes involved in maintaining cell–cell interactions (e.g., CAV2, JUP, NOTCH1, TJP2, DSC3, DSG3, ITGA9, ITGB4, and ITGB5) which may cause intercellular junctions to fail due to the inability to maintain strong adhesion between cells [51].

### 3.2. Mechanisms of Proteogenomic Signal Specificity and Apoptosis

**Figure 6:**
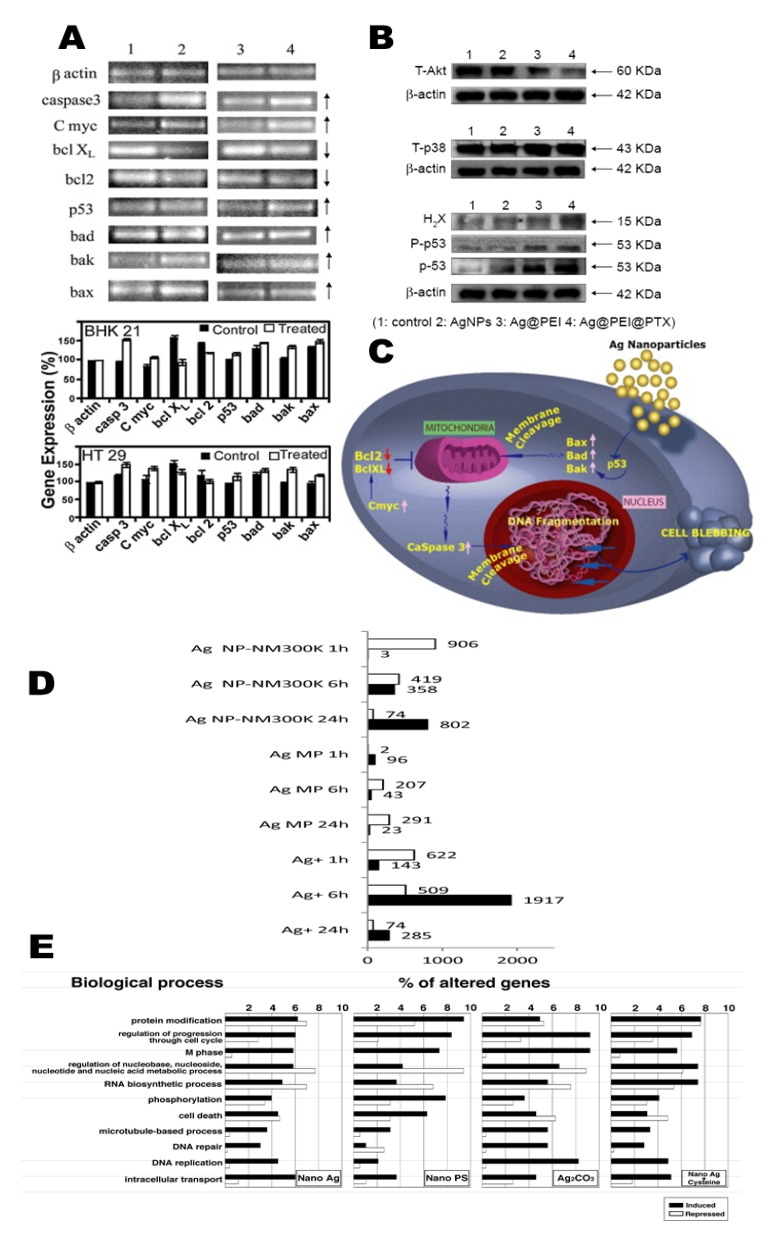
AgNPs inhibit the various proteins expression associated with NF-kB-based apoptosis. (**A**) Immunoblotting analysis of proteins, namely, p-NF-kB, BAD, Bcl-2, and Bax followed by densitometry of immunoreactive bands in 24 h A549 cells. Adapted with permission from Reference [102], Copyright 2010 Elsevier. (**B**) Microenvironmental apoptotic signaling pathway-related proteins are shown with complex Ag@PEI@PTX exposures in HepG2 cells. Intracellular apoptotic signaling pathway levels of the T-Akt pathway, T-p38 MAPK pathway, and activation of the p53 signaling pathway. β-actin acts as a control. Reprinted with permission from Reference [38], Copyright 2016 Dove Medical Press. (**C**) Graphic representation of AgNP-induced apoptotic protein expression. Adapted with permission from Reference [102], Copyright 2010 Elsevier. (**D**) Quantified and significantly altered genes in A549 cells after AgNPs, AgMPs (micro-sized particles), and Ag ions (Ag+) exposures delivered from various time scales. Adapted with permission from Reference [114], Copyright 2018 Royal Society of Chemistry. (**E**) 521, 188, and 298 genes were significantly modified through pure AgNPs, PS-NPs, and Ag_2_CO_3_ exposures, respectively. Every bar explains the ratio of genes categorized in each biological metabolism. Reprinted with permission from Reference [44], Copyright 2009 American Chemical Society.

Damage to the mitochondrial membrane potential (ΔΨm) affects the decrease, increase, and release of Bcl-2, BAX, and cytochrome c into the cytosol environment, respectively [101,102]. The expression of Bax, Bak, bad, P53, Bcl-2, Bcl X_L,_ caspase 3, and caspase 9 gene expression in A549 cells were studied with the pristine AgNPs, and drug-loaded AgNPs (Figure 6A,B). Here, P53 acts as the guardian of the genome and is encoded by the TP53 gene. This gene acts as a tumor suppressor and is either mutated or inactive in half of all cancer cells [52,56,85,103]. Accordingly, we also know that decreased Bcl-2 expression is influenced by the JNK pathway. c-Jun N-terminal kinases (JNK) is part of the mitogen-activated protein kinase (MAPK) family which is involved in apoptosis through the phosphorylation of Bcl-2, resulting in the deactivation of Bcl-2. The release of cytochrome c into the cytosol initiates a cascade that leads to caspase-3 activation through apaf-1 and caspase-9 [52]. The p21, p53, lamin B1, DNMT2, and metabolic activity in cells has significantly described which has long-term effects of AgNP-mediated epigenetic changes in tumor microenvironments. Depending on p53 and p21 mutations and the cellular context, AgNPs-mediated antiproliferative activity is different which may contribute to transcriptional suppression of tumor proliferation [87,88,104].

Many studies have widely reported that AgNPs are internalized within the microcellular environment, cell-to-cell interactions, and endosomes. Those internalized AgNPs affect the “Trojan-horse”-type mechanism which actively leads to cellular autophagy, apoptosis or necrosis [63,104]. For example, when AgNPs phagocytose into RAW 265 cells, active cells found in the culture medium, but not in death or damaged cells. Thus, AgNPs that are released from damaged cells into the culture medium promote the next biological response referred to as a Trojan-horse-type mechanism. The disappearance of AgNPs into cancer cells suggests that the NPs are ionized in the intercellular environment which results in functional damage. This was a valuable observation, phagocytosis of AgNPs can yield ROS, which initiates the acute systemic inflammation (i.e., TNF-α, IL-1β, IL-6, and IL-8 inflammatory signaling) [89]. The pro-inflammatory cytokines level of IL-1, IL-6, IL-8, IL-1 beta and cellular responses have been studied in the tumor microenvironment, which characterize the expression of CXCR1 and CXCR2 receptors of cancer cells. These act as indirect markers of inflammasome activation [89,105]. These acute and chronic systemic inflammation are affected by AgNPs, which may fail to stimulate several signaling pathways leading to tissue factor, inhibition of inflammatory signaling pathways, and apoptosis [90]. As per Gopinath et al. [102], major apoptotic signaling pathways are graphically presented in Figure 6C.

Furthermore, in the in vitro microenvironments, AgNPs exhibit anti-proliferative synergistic effects in a dose-dependent manner in human squamous cancer cells. The expression of the pro-apoptotic gene, Bax, was upregulated [53]. The molecular mechanism of non-cytotoxic AgNPs applied cell proliferation, the activity of the MAPK signaling cascade was measured for two reasons: The MAPK cascades have been involved in the regulation of cell proliferation in several physiochemical activities. MAPKs have been increased with high doses of AgNPs. Additionally, this study also revealed increased expression of p38pp, c-Jun, and c-Fos upon exposure to non-cytotoxic AgNPs which may suggest that the p38 MAPK pathway, via c-Jun and c-Fos is involved in the stimulatory effects of AgNPs in HepG2 cells [54]. Additionally, P-glycoprotein (Pgp) acts as an ABC transporter in the plasma membrane, which may develop the multidrug resistance (MDR) of cancer tissue. Inhibition of Pgp efflux activity proved to be dependent on the size of AgNPs. Pgp inhibition is associated with the molecular phenomena of drug-resistant breast cancer cells [55].

### 3.3. Fate of Stress-Related Therapeutics

Another cancer preventing pathway and redox transcription factor, nuclear factor erythroid 2-related factor 2 (Nrf2) is important in cancer cells. Nrf2 has a central role in saving cells from oxidative stress via antioxidant-responsive genes and genes of phase-II detoxifying enzyme metabolites. Nrf2, AP-1, and NF-kB are linked to many antioxidant functions [57]. Previous studies have shown that the proactive function of Nrf2 is lost by AgNPs in several cancer cells. Several types of NPs including AgNPs elevate heme oxygenase-1 (HO-1) expression in cancer cell proliferation and angiogenesis. Major studies on human HO-1 expression and Nrf2 knockdown have focused on human cancer and non-cancer cells. Nrf2-mediated gene expression seems to have failed [69,70]. Moreover, pristine AgNPs induced oxidative stress that triggered the Kelch-like ECH-associated protein 1 (KEAP1) redox switch which dissociates Nrf2 and it finally shifted to the nucleus [106]. Nrf2 influences cellular metabolism. Cytoprotective proteins (i.e., HO-1) has been influenced by Nrf2 activations. An enzyme of HO-1 counteracts cell death by producing Fe^2+^, biliverdin, and CO to stabilize ROS levels [107,108].

Figure 6D illustrates the overall molecular changes (transcript and metabolic) in comparison with various time points. However, heat shock proteins (HSP; HSP40, HSP60, and HSP70) are analyzed with AgNPs and applied to several stress conditions. These has been classified as a stress response in proteins from infections and inflammations [22]. The selective degradation of the HSP family or misfolded proteins is associated with oxidative stress. Kang et al. 2012, [57] showed the regulation of heme oxygenases (HMOX1, HMOX2) in the cancer microenvironment after AgNPs exposure, which may be linked to p38 MAPK signaling pathways [57,63].

VE-cadherin, an endothelial cell-specific adhesive molecule, plays an essential role in maintaining cell–cell junction stability, the structural maintenance of cells, and the prevention of barrier leakage [109]. AgNPs may stimulate mechanistic stress on VE-cadherin internalization that leads to homophilic impairments, actin rearrangements, and endothelial cell monolayer integrity [110,111]. In contrast, Ag+ ions exposure cannot induce the same damage. VE-cadherin internalization was triggered via extracellular AgNPs, which may lead to increased endothelial monolayer permeability and monocyte exudation [91]. Finally, increased LC3-I and LC3-II turnover is involved in essential autophagy gene pathways which was quantified after AgNPs exposure in HeLa cells. LC3 commonly acts as an autophagosome [92]. The catabolic process of autophagy in which unwanted or dysregulated waste segments has been separated into dual-membrane vesicles (autophagosomes) to undergo lysosomal degradation. Not long ago, several NPs induced autophagy leading to cell death and, puzzlingly, to increased cell survival [112,113]. Lastly, in Figure 6E, changes in genes involved in M phase, DNA repair, DNA replication, and cell death following exposure to AgNPs, PS-nanoparticles, Ag_2_CO_3_, and Nano Ag+ Cysteine have been classified [44].

## 4. AgNPs Currently Applied in Chemical Metabolomics and Suppression of Metabolic Pathways

Metabolomics (also referred as metabonomics or metabolic profiling) gives data from whole-cell responses to NPs which acts as valuable tools for small-molecule (<1500 Da) investigations. Metabolomic profiling of endogenous metabolites is widely used in the study of biochemical pathways (e.g., glycolysis to oxidative phosphorylation) in systems biology [93,115,116,117]. Metabolome (set of small molecules or metabolites) act as structural building blocks to transform biochemical reactions in every cell. High-resolution 1D and 2D NMR data are quickly used to extract differences in samples. However, clinical metabolic phenotypes (metabotypes) are different based on age, diet, race, gender, lifestyle, etc.

### 4.1. NMR Spectra and Assignment of Cancer Metabolites

**Figure 7:**
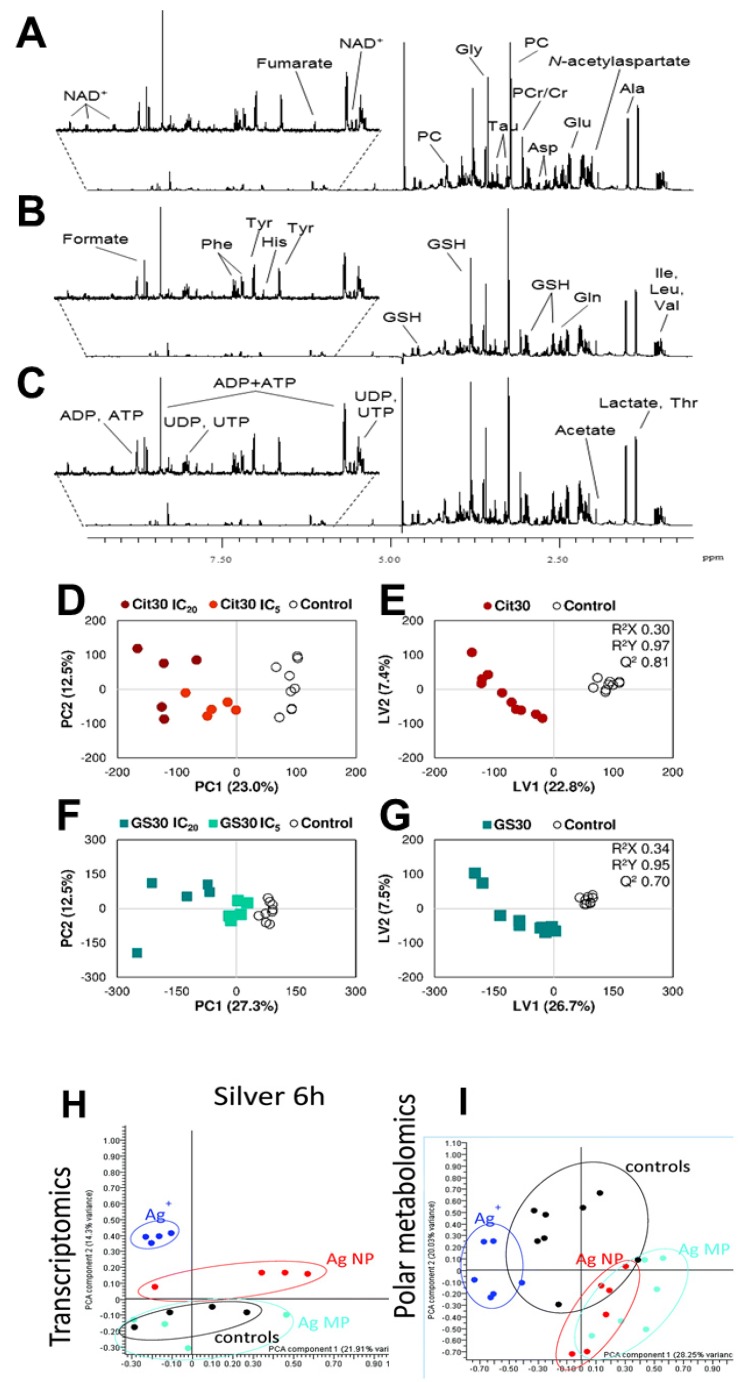
Solution-state NMR experiments: ^1^H variable spectral data of metabolites from AgNPs exposure in HepG2 cells, using a 5 mm probe: (**A**) controls, (**B**) Cit30 AgNPs, and (**C**) GS30 AgNPs. Multivariate statistical analysis of ^1^H-NMR spectral data from HepG2 control cells and cells exposed to (**D**,**E**) Cit30 and (**F**,**G**) GS30 AgNPs. (**D**,**F**) The PCA score plot. (**E**,**G**) The PLS-DA score plots produced by pairwise comparisons (control and AgNPs). Data revealed that cancer cells have high quality (i.e., Q^2^ = 0.8 and 0.7 for Cit30 and GS30 models, respectively). Abbreviations: Cit30, citrate-coated 30 nm AgNPs; GS30, green synthesis 30 nm AgNPs. Reprinted with permission from Reference [96], Copyright 2018 American Chemical Society. Pattern recognition of another cancer cells: PCA scores plots of (**H**) transcriptomics and (**I**) metabolomics data from A549 cells exposed to AgNPs, AgMPs, Ag^+^, and control. Explanations: control, black dot; Ag NPs, red dot; Ag MP, cyan dot; Ag+, blue dot. Adapted with permission from Reference [114], Copyright 2018 Royal Society of Chemistry.

Nanoparticles influence metabolite changes with a powerful analytical method, metabolomics, which gives a standard promising solution for metabolic pathway analysis [94,95]. The t_2_-weighted ^1^H-NMR spectral intensity of polar metabolites in HepG_2_ cells has shown in Figure 7A–C. All these ^1^H-NMR spectra were phase and baseline corrected. All the spectra were managed using either the “TopSpine” software (Bruker) or “VnmrJ” software (Agilent) packages. The signal of water and its nearby affected regions (between 4.33 and 5.50 ppm) were removed before analysis. The metabolite profile of IC_5_ concentrations of Cit30 AgNPs and GS30 AgNPs exposure in HepG_2_ cells highlighted with ^1^H variables. However, the targeted metabolite is marked which is consist of the sugars, amino acids, enzymes, and membrane. Aromatic spectral regions have been especially zoomed. Subtoxic concentrations such as IC_5_ and IC_20_ of AgNPs exposure has decreased the energy consumption in cancer cells [96].

Under physiological conditions, the most common way to generate energy in cancer cells is glycolysis; thus, glucose scarcity forces the metabolic switch back to oxidative phosphorylation (so-called the Warburg effect) [118]. These metabolic switch phenomena have been observed in a variety of experimental setups, ranging from yeast to mammalian cell lines [119,120]. At non-toxic concentrations AgNPs were shown to induce suppression of the TCA cycle intermediates, inhibition of oxidative phosphorylation with a concomitant decrease of ATP production and increased glycolysis [121].

### 4.2. Pattern Recognition of Cancer Cells

Spectra were normalized by whole spectra area and aligned which minimize chemical shift variations. Metabolites can be discovered through NMR-based metabolomics by detecting characteristic spectral patterns, for example, healthy vs diseased. Principle component analysis (PCA; Figure 7D,F,H,I) and particle least squares discriminant analysis (PLS-DA; Figure 7E,G) subjected to 1D ^1^H-NMR spectra of extracts from cancer cells which differentiate the clear separation between control and AgNPs [114]. A PCA represents basic discrimination. To get better separation, supervised methods of PLS-DA were performed to explore the differences in metabolomes among the groups. They are useful tools to extract differences in NMR data. These approaches provide higher accuracy of inter and intra group variances. A profile of control cells and Cit30 or GS30 AgNPs exposure in HepG2 cells has described statistical variations among AgNPs from control samples. A PCA acts as a primary method but PLS-DA is more robust (Q^2^ = 0.8 and 0.7 for Cit30 and GS30 score plots, respectively) in samples by univariate and multivariate investigations (Figure 7D–G). The validity of the model was assessed according to cross validation results. Additionally, corresponding loading delivered several metabolic regulations by AgNPs [96,114].

### 4.3. Topology of Energy Metabolism

When glucose concentration is not modified under AgNPs treatment, pyruvate and TCA cycle activity intensify by increasing pyruvate intake from the culture medium, along with down-regulation of intracellular levels of anaplerotic amino acids such as glycine, N-acetyl aspartate, aspartate, glutamate, and polyunsaturated fatty acids, reflecting their energy invention via oxidation process [97,122]. The cellular ATP amounts are increased in line with this hypothesis. In contrast, it has also been reported that HepG2 cells (and other cell types) treated with noncytotoxic doses of AgNPs (up to 8 µg/mL) decrease intracellular ATP [97]. Previous studies have also shown that ATP loss was linked to a shift in energetic metabolism from oxidative phosphorylation and fatty acid oxidation to glycolysis based on cellular lactate, pyruvate, cholesterol, and triglyceride levels [94].

Pyruvate acts as an energy substrate and aids NAD+ regeneration via conversion into lactate [123]. Moreover, LDH acts as cytoplasmic enzyme metabolite that is essential for cell duration. Cells treated with AgNPs for 24 h showed a significant rise in LDH release relative to natural cells. The intensity of LDH leakage was significantly higher equated to controls or single treated cells [85]. In AgNPs-induced metabolic activity in lungs, downregulation of glycolysis (decreased lactate) and TCA cycle (decreased succinate and increased TCA cycle substrates alanine and glutamate) was significantly impaired. In this way, the metabolic activity of various organs was determined in mice [85]. The Nrf2 canonical pathway was significantly influenced by AgNPs, resulting in the downregulation of antioxidant defense mechanism. This resulted in a significant alteration of protein ubiquitination.

Similarly, another study found that more pyruvate in neuronal cells reduced the toxic effects induced by zinc ions thereby mitigating ATP loss and cell death [121]. Downregulated TCA cycle activity, energy depletion, increased glutaminolysis, and GSH-based antioxidant protection has been reported in HaCaT cells [98,124]. In in vivo environments, the liver is mainly responsible for pyruvate recycling (produced from lactate by other organs) and gluconeogenesis. Particularly, low ATP yield by AgNPs exposure in glioblastoma cells and human fibroblasts is linked to mitochondrial dysfunction and structural damage caused by deposition of nanoparticles or oxidative stress [76].

Furthermore, it has been proven that AgNPs can directly inhibit the activity of mitochondrial ATPase in liver, thus reducing ATP production [125]. Additionally, AgNPs have been impaired in oxidative phosphorylation metabolism in the mitochondrial microenvironment [126,127]. The creatine and phosphocreatine have significantly decreased, which mainly reflecting energy storage and their transportation. Same study has found in HepG2 cells treated by graphene nanosheets [128].

### 4.4. Topology of Antioxidant Defense Metabolism

An important oxidative stress metabolite of GSH has widely increased in intracellular microenvironment which is key player in defense systems [129]. Another notable result agrees where HepG2 cells incased GSH levels (1.1-fold), along with SOD activity. Overall, around 10% cell viability (survival rate) has decreased by AgNPs exposure [130,131]. Also, the cytotoxicity was delivered to induce diminished GSH levels which may reflect an oxidative stress [99,132,133]. Also, citrate coated AgNPs-treated cells expressed significantly increases methionine and histidine. Amplified those amino acids was metabolically stressed in tumor microenvironment [134].

**Figure 8:**
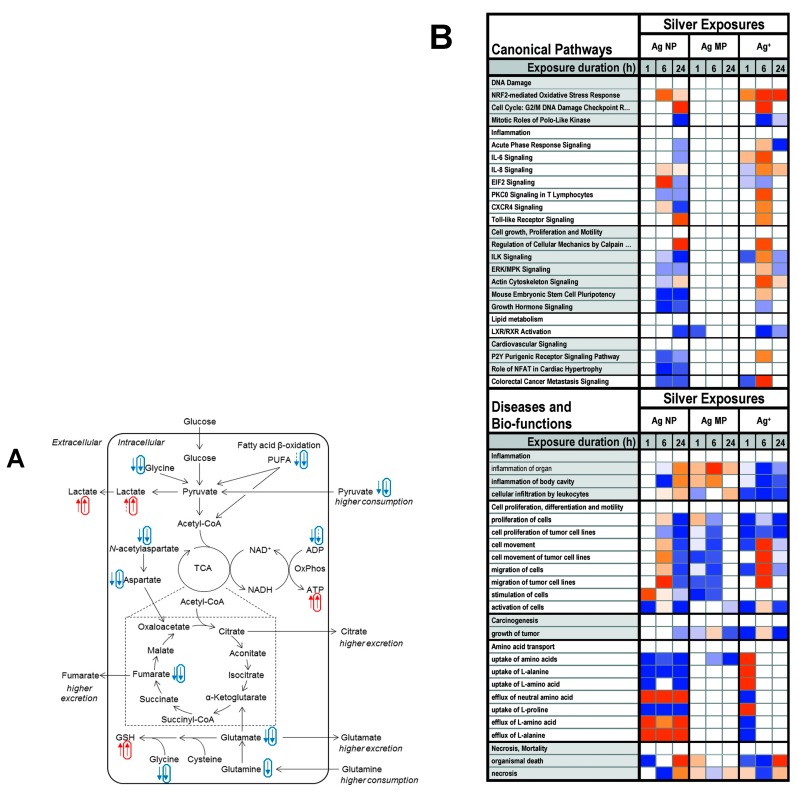
(**A**) Metabolic impacts of AgNPs exposure on energy changes (glycolysis and the TCA cycle) in cancer cells. As equated with controls, normal and rounded arrows signify effects caused, separately by Cit30 AgNPs and GS30 AgNPs. Spoiled symbols indicate <10% difference; dense symbols indicate >50% difference. Reprinted with permission from Reference [96], Copyright 2018 American Chemical Society. (**B**) The top 20 canonical pathways and top 20 diseases and bio-functional metabolism summarized with the AgNPs, AgMP, and Ag+ exposures in A549 cells. The metabolic activity was calculated by ingenuity pathway analysis (IPA), indicating predicted pathway activation (orange) or inhibition (blue). The limit of color intensity was settled to a z-score ≥ 2. Adapted with permission from Reference [121], Copyright 2018 Royal Society of Chemistry.

The PVP-functionalized AgNPs delivered autophagocytotic responses in HepG2 cells via size-dependent manner, even at relatively low or noncytotoxic concentrations. This AgNPs treated autophagocytic responses acted as a cytoprotective mechanism at subtoxic doses in HeLa and HepG2 cells. While the amino acids upregulated by citrate coated AgNPs-treated cells (possibly reflecting protein catabolism), GS30 decreased intracellular glutamine levels, may formed glutaminolysis activity [135,136]. The cellular metabolome, metabolic adaptations within several pathways such as glycolysis and TCA cycle are proposed in Figure 8A.

### 4.5. Topology of Membrane Metabolism

Phosphocholine (PC) and phosphatidylcholine (PPC) are involved in the maintenance of cellular membranes which are synthesized mainly from the CDP–choline pathway and a section of the Kennedy pathway. This CDP–choline pathway starts with the intake of exogenous choline into the cell and converts PC from choline phosphorylation. Following treatment of AgNPs to cells, a negative correlation between choline intake and PPC was found, and it could reveal the breakdown of phospholipid synthesis [137]. The metabolic effect was particularly noticed in green synthesis (GS30 AgNPs) and citrate 30 nm AgNPs-treated HepG2 cells, where significant correlation was found among many metabolites in CDP–choline pathways. Additionally, in GS30-treated HepG2 cells, intermediate metabolites of this pathway have delivered solid correlation with the uriadine-5′ triphosphate (UTP) which may spark their role as a cytosine 5′-triphosphate (CTP) precursor. Additionally, cholesterol esters are amplified in cells which could be connected in the form of biological detoxifications [99,137,138].

From previous studies, GSH and ATP were significantly reduced. Likewise, amino acids have been minimally extended, and in opposition, creatine and phosphocreatine remained unaffected. The cholesterol esters of polyunsaturated fatty acids and PPC have been decreased [139,140]. On the contrary, from the previous literature, AgNPs- and Ag+-treated metabolome signatures delivered plenty similar landscapes, although a few variances were noted. Mainly, amino acid were highly increased but less notably increased in ATP and GSH in Ag^+^-treated cells.

Figure 8B shows the top 20 canonical pathways and the top 20 “diseases and bio-functions” associated with the silver exposures, ordered by function or process from the IPA’s pathway activity analysis function, representing predicted pathway activation or inhibition. Particularly prominent for silver was the Nrf2-mediated oxidative stress response pathway, predicted to be activated by Ag NP at 6 h and by Ag+ at all timepoints [121]. Additionally, the Nrf-2 signaling pathway was significantly disturbed by all test chemicals, resulting in the downregulation of antioxidant defense proteins and, consequently, increased ROS levels. This resulted in a significant alteration of protein ubiquitination are possibly increased toxicity. The eIF2 signaling pathway was modified after the AgNPs and Cd^2+^ exposures, owing to the downregulation of ribosome subunits and proteins involved in translation initiation [141]. Collectively, microenvironmental oxidative stress has settled in several locations. These findings highlight the therapeutic potential of using high-throughput omics technologies, potentially helping to reduce the demand for highly efficient in vivo delivery.

## 5. Conclusion, Challenges, and Future Perspectives

Significant developments have been made in the field of AgNPs-based cancer genomic profiling over the past two decades. This review aimed to deliver a comprehensive view of AgNPs for diagnostics and therapeutic treatment of several cancer genomes and metabolomes from multi-omics technology (i.e., genomics, transcriptomics, proteomics, and metabolomics). The multi-omics sciences have globally matured to target structural, molecular, and phenotypic changes in systems biology [142,143]. Here, we have carefully accounted for the variability of several genomes, proteomes, and metabolomes which are mostly highly listed in terms of AgNPs-based targeting therapy in tumor microenvironmental locations. The use of AgNPs with fluorescent imaging provides anti-proliferative agents and promotes targeted drug delivery and the ability to cross membrane barriers. In addition, this mechanotransduction of AgNPs has secured an important place in anticancer activity implementation. Like this, functionally modified, PDT-doped AgNPs are currently used for tumor diagnosis and treatment. Now, researchers have found many ways to kill cancer cells, but commercial translation using nanomedicine has yet to happen.

Moreover, we have specifically summarized recent progress in genes/proteins (i.e., tumor suppressor genes: P53, P21, P27; apoptosis genes: Bcl-2, BAX, TNF-α, LC3-I, LC3-II; protein folding and assembly genes: HSP60, HSP70; oxidative stress genes: SOD1, SOD2, GPx-1; cytokine activity genes: IL-1beta, IL-8, etc.) after AgNP exposure. Similarly, targeted and quantified metabolites (i.e., energy: ADP, ATP, glucose, lactate, pyruvate; antioxidant defense system: GSH; neurotransmission: glutamine, glutamate; catalytic activity: LDH; surfactant: Pcho, GPcho; fatty acid: triglyceride; cholesterol; amino acids: alanine, valine, phenylalanine; second messenger: myo-inositol) are carefully for accounted in regard to their changes in tumor environments. The biochemical changes of metabolites are associated with LDH catabolism and amino acids catabolism. This amino acid catabolic activity of AgNPs may be helpful in terms of diagnostics. These in vitro deliveries highlight the therapeutic potential in specific genes to alter stem cell pathways. The enhanced genome in different cancer cellular microenvironment has been carefully addressed in this review.

For a cancer-targeting chemical metabolomics therapy to be effective, highly efficient metabolite delivery is necessary. Study of this molecular clinical biomarker was investigated in tumor microenvironments with different culture models (e.g., 2D and 3D methods). Finally, the metabolic-mediated reduced glycolytic activity or decreased levels of energy-related metabolites, amino acids catabolism, neurological behaviors, and antioxidant defenses changes have been found in many cancer experiments [137]. Understanding AgNPs-induced cancer genome landscape from several in vivo and in vitro studies has been helpful for therapeutic applications in human cancer cells. The identification of biomarkers and the development of nanomedicine are exciting areas of research in mechanobiology [144,145,146]. In recent times, cellular targeted bioimaging has required bright nanoparticles which are non-toxic to cells. More promising imaging techniques are also required to understand the molecular diversity in tumor suppressor proteogenomic.

With the outcome of these studies, AgNPs and nano-biohybrid AgNPs have displayed anticancer effects by either direct or indirect action on tumor cells, both in vitro and in vivo. AgNPs dispersion capacity in PBS and colloidal solution play important roles in biological systems. More importantly, pure and nano-biohybrid AgNPs-based results vary according to the NPs’ structure, surface tension, stability, sensitivity, agglomeration, size, dose, pH, and experimental periods [147]. In cancer cell microenvironments, AgNPs enhanced ROS radicals such as ^•^OH, O_2_^•-^, and ^1^O_2_. These free radicals can remodify the microenvironmental metabolic reactions in the cytoplasm, mitochondria, and nucleus as well as the effects of the electron transport-chain that triggers apoptosis [148,149]. At present, molecular techniques, such as the LDH-assay, MTT-assay, Western blotting, and real-time PCR, are dynamically promising to ensure proteogenomic imaging which might be necessary for medicinal efficiency. More investigation is required to further study AgNPs delivery, biocompatibility, and cytotoxicity of regular muscles.

Thus far, scientists have not yet reached the era of personalized medicine using AgNPs. However, strong progress has been and continues to be made. Overall, proteogenomic and chemical metabolomics in cancer is a dynamic process. Here, the mechanotherapy of AgNP-based cell therapies can be exploited to generate several chemo modulations against solid tumors. These high-throughput therapies against the tumor microenvironment can be further studied in clinical studies and may lead to the development of therapies that are applicable to a variety of cancer cell types. Clinical therapy using AgNPs is promising and needs to be extensively studied in the future. Simultaneously, healthcare concerns regarding therapeutic applications of AgNPs must also be addressed.

## Figures and Tables

**Table 1:** Summary of molecular effects on cancer growth controls due to the physicochemical properties of AgNPs upon exposure in in vitro methods.

Year	Cancer Cells	Size (NM)	Concentration	Experimental Time	Mechanobiological Outcomes	Reference
2009	Human hepatoma cells (HepG2)	7–20	Up to 250 mg/mL	24 h	Increased glutathione (GSH) level; increased apoptosis (AO-EB staining) and caspase 3 activity	[6]
2009	A431HT-1080	7–20	1.56–500 μg/mL	24 h	Apoptosis induced in both A431 and HT-1080 cell lines	[20]
2009	Human lung adenocarcinoma (A549 cells) THP-1 monocytes	30–50 69	Up to 15 mg/mL	24 h	ROS generation: formation of DNA adducts Cytotoxicity; ROS; oxidative stress	[22]
2013	Human lung carcinoma (A549 cells)	<100	0, 25, 50, 100, or 200 µg/mL	24, 48 h	Intracellular ROS levels; cell cycle; proliferating cell nuclear antigen (PCNA) protein expression level	[23]
2011	Human colon cancer cells (HT29)	172.6 ± 27.1	12–48 μg/mL	24 h	ROS generation; induced apoptosis (AO-EB staining and annexin V staining); increased DNA fragmentation and expression of caspase 3; mitochondrial membrane depolarization	[26]
2009	Human hepatoma cells (HepG2)	5–10	Up to 2 mg/mL	24 h	ROS generation and change of oxidative stress-related gene expression (catalase, GPx1, MT1b, and SOD1) Detection of g-H2AX phosphorylation; lactate dehydrogenase (LDH) leakage	[39]
2015	Human breast cancer cells (MDA-MB-231)	300 and 600	5–25 µg/mL		Targeting p53; Bcl-2; p-p53; ROS; anticancer effects	[43]
2009	Human hepatoma cells (HepG2)	7–10	1 mg/mL	24 h	Increased micronucleus formation	[44]
2009	PC12 cells	200	2.5–25 µg/mL	24 h	Gene expression of Gss; Gpx; Txnrd	[45]
2008	Mouse fibroblast (NIH3T3) and Human colon cancer cells (HCT116)	1–100	Up to 100 mg/mL	72 h	ROS generation; increased apoptosis (annexin V staining); expression of p53 and c-Jun N-terminal kinases (JNK) activation. NIH3T3-induced release of cytochrome c into the cytosol and translocation of Bax gene to mitochondria and decreased Bcl-2 expression	[46]
2011	Human hepatocyte cell line (L02)	37.8, 6.7	Up to 100 mg/mL	24 h	Increased MDA formation and reduced GSH level and SOD activity; induced DNA breakage (comet assay)	[47]
2010	Human hepatoma cells (HepG2)	5.9–3.3, 23.8–6.7, 47.5–22.1	Up to 50 mg/mL	24 h	ROS generation; induced apoptosis (annexin V staining and Hoechst 33342 staining) Increased and decreased GSH level; reduced SOD activity; cell cycle arrest in S phase	[48]
2014	human colon carcinoma cells	10, 20, 40, 60, and 100	0 to 10 µg/mL	24 h	Intracellular ROS levels; IL-8 release; mitochondrial activity; cell viability; cell proliferation	[49]
2009	Human carcinoma cell line (HeLa S3)	2–5	Up to 120 mg/mL	3, 4 h	Increased expression of HO-1 and MT-2A expression; induced apoptosis (annexin V staining)	[50]
2016	Human colorectal carcinoma cell line (T84 cells)	10, 20, 75, and 110	20 and 100 mg/mL	48 h	Genes related to cell-cell junctions and epithelial barrier functions	[51]
2017	Human cervical cancer cells (HeLa CCL2)	420	20–100 μg/mL	24 h	Proapoptotic gene expression, including *P53,* P21, *BAX,* BAK, CASP3, and CASP9,	[52]
2016	Human tongue squamous carcinoma (SCC-25)	10	0.31 to 10 g/mL	48 h	Reduced proliferation and viability; cytotoxicity; cell cycle arrest; cell morphology analysis;	[53]
2014	HepG2 cells	10 and 100	2.0 and 4.0 mg/L	24 h	Non-cytotoxic doses induced p38 MAPK pathway activation and led to the promotion of HepG2 cell proliferation	[54]
2019	Human breast adenocarcinoma (MCF-7 cells)	5, 75	212 µM	24, 48 h	ER stress markers; ER calcium levels	[55]
2018	Human lung adenocarcinoma (A549 cells)	633	10, 40 μg/mL	24, 48 h	Bcl-2; Bax; caspase-3, 7 expression	[56]
2012	SK-OV3 cells	7.5 ± 2.5	1, 5, or 10 μg/mL	24 h	Nrf2 knockdown cells; DNA damage and apoptosis	[57]

**Table 2:** In cancer microenvironment, targeted therapeutic biomarkers (genome and metabolome) in different cancer cells upon exposure to different sizes of AgNPs in in vitro methods.

Omics	PDBe-KB/KEGG	Gene/Proteins	Potential Outcomes	References
**Genomics and Proteomics**	P42574, Q13490, Q8IH92	Caspase-3, 9; T-Akt; T-p38; P-53	Reponses to hypoxia B cell homeostasis Proteolysis	[20,37,38]
	Q07817	Bcl-2	Cell morphogenesis Ossification; apoptosis regulator	
	Q8ISJ0, Q07449, P07203	SOD1, SOD2, GPx-1	Oxidative stress-related genes Familial amyotrophic lateral sclerosis (ALS) by mutation SOD1	[39]
	P61604	HSP60, HSP70 (heat shock proteins)	Protein folding and assembly Prevention of aggregation of unfolded protein chains	[41,42]
	Q07812	BAX	Apoptosis process Cell death	[56]
	P06762, A8JBZ0	HMOX1, HMOX2	Heme catabolism Heme oxygenase family	[63,70,72]
	Q8CHP8	NA/K ATPase; Pgp; EDEM	Plasma membrane protein	
	Q16611, Q38998, P04049	Bak, Bax, Bid, Bcl-2, p53, Caspase-3, 6, 9; NF-kB, P38, AKT1, RAF, MEK,	Proapoptotic and antiapoptotic genes expression Genomic damage Signaling pathways of cell survival Cytotoxicity Apoptosis-regulated genes	[85]
	Q9W1M7, P49137	PI3K, AKT, P38MAPK signaling cascade	Protein Synthesis Cell proliferation Signaling pathways responses Acts as molecular sensor Small molecule inhibiters	[86]
	Q8IH92	Tumor protein P53 (TP53), P21, P27	Tumor suppressor gene Apoptosis Gene therapy	[87,88,89]
	P10145, O73909	IL-8, IL-1beta	Cytokine activity Immune regulation responses	
	P01375	TNF-α	Inflammatory signaling Apoptosis	[90]
	Q8AYD0	VE-cadherin; p-VE-cadherin	Calcium ion binding Junctional stability	[91]
	Q38998	P-AKT; AKT; LC3-I; LC3-II	Signal transduction Autophagic cell death Autophagosomes	[92]
	Q9BYN0	SRXN1 (sulfiredoxin 1)	Antioxidant protein	[93]
	Q16236, P09601	Nrf2; HO-1	Cytoprotection	
	C00031, C00186, C00158	Glucose, lactate, citrate	Energy cycle	[39,84,87,94]
	C00157, C00670	PhosphocholineGlycerophosphocholine	Energy storage Membrane stabilizer Surfactant; Emulsifier	
	K00016	LDH leakage	Catalytic activity	
	C00051	Glutathione	Antioxidant defense systems	
	C00385, C00262	Xanthine; hypoxanthine	Tissue hypoxia	
	C01104, C00051	Trimethylamine N-oxide; GSH	Stabilizer	[95]
**Metabolomics**	C00008, C00002, C00003	ADP, ATP, NAD+	Energy generation	[96]
	C00422, C00187	Triglyceride; Cholesterol	Fatty acids, fat(lipids) Contributing the cell walls	[97]
	C00022, C0004, C00122	Pyruvate, succinate, fumarate,	TCA cycle intermediates	[98]
	C00041, C00183, C00079	Alanine, valine, phenylalanine,	Amino acids	
	C00064, C00025	Glutamine, glutamate,	Neurotransmitter metabolites	
	C00137	Myo-inositol	Second messenger	
	C00051, Q8ISJ0	GSH, SOD; lipid peroxidation; LDH	Stabilizer	[20,99]

PDBe-KB, Protein Data Bank in Europe - Knowledge Base; KEGG, Kyoto Encyclopedia of Genes and Genome.

## Abbreviations

Ag	Silver
Ag2CO3	Silver carbonate
AP-1	Activator protein 1
ATP	Adenosine triphosphate
Ca2+	Calcium ions
Cit	Citrate
Conc	Concentrations
CXCR1	C-X-C Motif Chemokine Receptor 1
DNA	Deoxyribonucleic acid
GS	Green synthesis
GSH	Glutathione
h	Hours
H_2_O_2_	Hydrogen peroxide
IL	Interleukin
JNK	c-Jun N-terminal kinases
LDHQ	Lactate dehydrogenase
mg/mL	Milligram/milliliter
MTT	Methylthiazolyldiphenyl-tetrazolium bromide
NAC	N-acetylcysteine
NAD+	Nicotinamide adenine dinucleotide
NADP+	Nicotinamide adenine dinucleotide phosphate
NF-kB	Nuclear factor kappa-light-chain-enhancer of activated B cells
nm	Nanometer
NMR	Nuclear magnetic resonance
NPs	Nanoparticles
OPLS-DA	Orthogonal PLS-DA
OS	Oxidative stress
p38 MAPK	p38 mitogen-activated protein kinases
PCA	Principal component analysis
PCR	Polymerase chain reaction
Pgp	P-glycoprotein
PLS-DA	Partial least squares-discriminant analysis
Ref	References
RNA	Ribonucleic acid
ROS	Reactive oxygen species
SOD	Superoxide dismutase
SRXN1	Sulfiredoxin 1
TCA	Tricarboxylic acid
TNF	Tumor necrosis factor
µg/L	Microgram/Liter
μm	Micrometer
